# Effectiveness of *Lactiplantibacillus plantarum* in enhancing the folate content of *injera* made with different cereals

**DOI:** 10.1002/fsn3.3560

**Published:** 2023-07-19

**Authors:** Aynadis Tamene, Tesfaye Mekuriyaw, Kaleab Baye

**Affiliations:** ^1^ Center for Food Science and Nutrition Addis Ababa University Addis Ababa Ethiopia; ^2^ Addis Ababa Technical and Vocational Training and Technology Bureau Kolfe Industrial College Addis Ababa Ethiopia

**Keywords:** cereals, fermentation, folate, injera, *L. plantarum*

## Abstract

Fermentation can contribute to the supply of essential vitamins like folate, but studies exploring this potential are scarce. Injera is an Ethiopian fermented pancake‐like flatbread made from different cereals. The study aimed to investigate the effect of injera‐making process using different cereals (tef, sorghum, wheat, and barley) on folate content and to evaluate the effectiveness of *Lactiplantibacillus plantarum* in enhancing folate of injera made with different cereals. Cereals were used alone or in combination (tef and sorghum (1:1), wheat and sorghum (3:1), sorghum (100%), and barley (100%)). *L. plantarum* previously isolated from tef dough and ersho (fermentation batch) collected from the households were used as starters. Folate content of the flour, dough, and injera was determined by microbiological assay. Contribution of consumption of injera made with different cereals to the folate requirement of children and women of reproductive age was evaluated. Among the studied cereals, the highest average folate content (49.9 μg/100 g) was observed in 100% sorghum flour and the least (32.2 μg/100 g) in 100% barley flour, on dry weight basis. After fermentation, the highest average folate content (60.1 μg/100 g) was observed in 100% sorghum dough fermented with *L. plantarum*. Highest average folate content (15.45 μg/100 g) per fresh weight was observed in wheat and sorghum (3:1)‐blend injera fermented with *L. plantarum*. Consumption of *L. plantarum*–fermented injera made with different cereals contributed up to 8% of the recommended folate intake of women of reproductive age.

## Graphical abstract

- *Lactiplantibacillus plantarum* strain can be used as a potential folate source not only for injera made with tef but also for any injera that could be made with other cereals.
- Consumption of any injeras fermented with *L. plantarum* contributed much more to folate RNI of children and women of reproductive ages than consumption of injera fermented with ersho.

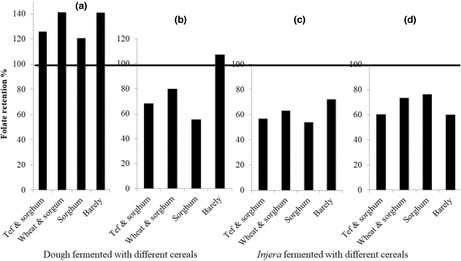

## 1 INTRODUCTION

Folate (vitamin B_9_) plays a key role as an essential coenzyme in organisms to provide one‐carbon units for nucleotide biosynthesis, amino acid metabolism, and DNA methylation (Crittenden et al., 2003; Iyer & Tomar, 2009; Rossi et al., 2011). Folate is a crucial component in maintaining the individual's health over the course of life (Iyer & Tomar, 2009). Folate is also needed to prevent neural tube defects (NTDs) in the developing fetus. Inadequate intake of folate leads to folate deficiency (Herrmann & Obeid, 2011a).

Folate deficiency is prevalent in many countries (McLean et al., 2008; Youngblood et al., 2013). In Africa, folate deficiency is mainly related to poor dietary diversity and reduced food intake (Dewey & Brown, 2003). The deficiency of folate has been implicated in a wide variety of disorders such as Alzheimer's disease, coronary heart diseases, osteoporosis, increased risk of breast and colorectal cancer, poor cognitive performance, hearing loss, and NTDs (LeBlanc et al., 2007, 2011). Inadequate maternal folate status has also been associated with low infant birth weight, preterm delivery, and fetal growth retardation (Scholl & Johnson, 2000). Megaloblastic anemia and elevated blood concentrations of homocysteine have also been linked to folate deficiencies (Aslinia et al., 2006; Ho et al., 2011). Insufficient folate intake is also reported to be associated with high blood homocysteine concentration, a potential risk factor for coronary heart disease and stroke (Djuric et al., 2008). Consequently, mandatory folic acid fortification has been implemented in more than 60 countries around the world (Crider et al., 2011).

On the other hand, very high folic acid intake has been associated with high concentrations of unmetabolized folic acid in the blood, which has been associated with the growth of preneoplastic lesions, cancer, and cognitive impairments (Herrmann & Obeid, 2011b; Hoekstra et al., 2008; Hooijberg et al., 2004, 2006; Morris et al., 2010). Besides, high daily folic acid consumption could mask and delay the diagnosis of an underlying vitamin B12 deficiency and thereby allow vitamin B12 deficiency‐associated neuropathies to progress (Institute of Medicine, 2000). Although the natural form (folate) is safer relative to the synthetic form (folic acid), animal‐source foods and selected dark‐green leafy vegetables are dietary sources; regular consumption of these foods is limited in low‐ and middle‐income countries (LMIC) (Zerfu et al., 2016). This along with the low availability of folic acid‐fortified foods leaves behind a significant proportion of the population living in LMIC to be at a greater risk of folate deficiency and its adverse effects (Haidar, 2010; McLean et al., 2008; Taye et al., 2015).

In recent years, the natural fortification of folate in fermented foods by the introduction of microbes that produce folate has attracted attention. Lactic acid bacteria (LAB) and yeasts, which are the main actors of fermentation, could produce up to 100 μg/L and 145 μg/g cell mass, respectively (Hjortmo et al., 2005; Iyer & Tomar, 2009; Sybesma et al., 2003). Up to 256 μg /L total folate production to the growing medium by genetically engineered *L. lactis ssp. lactis* was also reported by Sybesma et al. (2003).

Cereal‐based foods are widely consumed as the primary staple foods in many countries of the world, especially in Africa (Tamene, Kariluoto, et al., 2019). Many cereal‐based staples typically undergo a fermentation step before consumption (Humblot & Guyot, 2008). For example, *injera* is a leavened, flat, and round cereal‐based fermented bread made from different cereals (Mengesha et al., 2022). It is a staple food that is widely consumed in Ethiopia (Baye et al., 2013).

*Injera* in Addis Ababa (Ethiopia's capital) is preferably made with tef (*Eragrostis tef*)—an ancient pseudo‐cereal that has its center of origin and diversity in Ethiopia (Baye, 2014). A recent study investigated the effect of *injera* processing (fermentation and baking) on tef *injera* samples collected from Addis Ababa (Tamene, Kariluoto, et al., 2019). In a subsequent study, a folate‐producing *Lactiplantibacillus plantarum* P2R3FA was isolated from tef fermentation and it was successful in increasing the folate content of *injera* made with tef (Tamene, Baye, et al., 2019; Tamene et al., 2022). However, whether similar folate‐producing effects can be expected for *injera* made from the fermentation of other cereals remain unknown.

According to Baye et al. (2013), while *injera* is preferably prepared from tef in major cities, tef‐ and sorghum‐based *injera* are commonly consumed in the lowlands, whereas barley and wheat *injera* are common in the highlands of Ethiopia. Therefore, this study aimed to investigate the effect of *injera* preparation using different cereals on folate concentration. In addition, it investigated the possibility of enhancing the folate content of *injera* made with different cereals using *L. plantarum* P2R3FA as a starter isolated from prior tef fermentation.

## 2 MATERIALS AND METHODS

### 2.1 Chemicals and raw materials

Unless otherwise specified, all the necessary chemicals and reagents used in this work were purchased from Sigma‐Aldrich Chemie GmbH (Buchs, Switzerland). The raw materials (tef, wheat, sorghum, and barley) were obtained from the local market of Addis, Ethiopia.

### 2.2 Sampling and sample preparation

Information on the different types of cereals used for *injera* making was obtained from the literature. The most commonly used grain types and mixing ratios for *injera* preparation were taken from Baye et al. (2013) and actual observations of selected households.

Accordingly, cereals such as sorghum (*Sorghum bicolor*) “Melkam” variety, tef (*Eragrostis tef* (Zucc.) Trotter) “Quncho” variety, wheat (*Triticum aestivum*) “Beka” variety, and barley (*Hordeum vulgare*) “Hb 1969” variety were selected for the current study. The blending ratios used in making the *injera* were as follows: sorghum alone (100%), sorghum and tef (1:1), wheat and sorghum (3:1), and barley alone (100%) (Table 1). Different cereal samples were collected from a local market in Addis Ababa. A 5 kg sample of each cereal was purchased and transported to a laboratory at the Center for Food Science and Nutrition of Addis Ababa University. The different cereals went through different stages of preparation; Tef was cleaned (low‐quality grains and dust such as wood chips and other unwanted materials were removed using a sieve) and milled using a disc mil (RUNMAO Machinery, Model number160, China). Sorghum was also cleaned, manually dehulled, and milled. Wheat was also cleaned and milled. Barley was cleaned, conditioned (soaked in cold water for 15 min), dehulled, and milled. Flours were blended using a dry particulate blender (MXBAOHENG 30L, Dry Powder Mixer, China) and subjected to four experimental runs (Table 1).

**TABLE 1:** Blending ratio used for the preparation of *injera.*

Experimental run	Blends of cereal flour
1	Tef: Sorghum (1:1)
2	Wheat: Sorghum (3:1)
3	Sorghum (100%)
4	Barley (100%)

### 2.3 Preparation of *injera* made with *L. plantarum* and *ersho* as inoculums

#### 2.3.1 Preparation of the inoculums

Two different inoculums were prepared. A leftover from previous successful spontaneous fermentation batch (*ersho*) which was taken from household was used as inoculum to prepare traditional *injera*.

The folate‐producing *L. plantarum* P2R3FA, previously isolated from fermented tef batter (Tamene, Kariluoto, et al., 2019), was cultivated by streaking the strain conserved at −80°C in De Man, Rogosa, and Sharpe (MRS) broth and glycerol 40% (v/v) on MRS agar and incubated at 30°C for 48 h. A colony was collected from each pure culture plate, grown in MRS broth (24 h, 30°C), and centrifuged (14,000× *g*, 7 min). The pellets were washed twice with the same volume (9 mL) of sterile saline solution (0.9% NaCl) and resuspended in the same volume of solution. The final suspension contained around 10^9^ colony‐forming units (cfu)/mL. The inoculum for the fermentation of *injera* with the folate‐producing *L. plantarum* P2R3FA was prepared by mixing this suspension with each of the respective cereal flour (1:1) (v/w).

#### 2.3.2 Preparation of *injera* made with different cereals and inoculums

**FIGURE 1:**
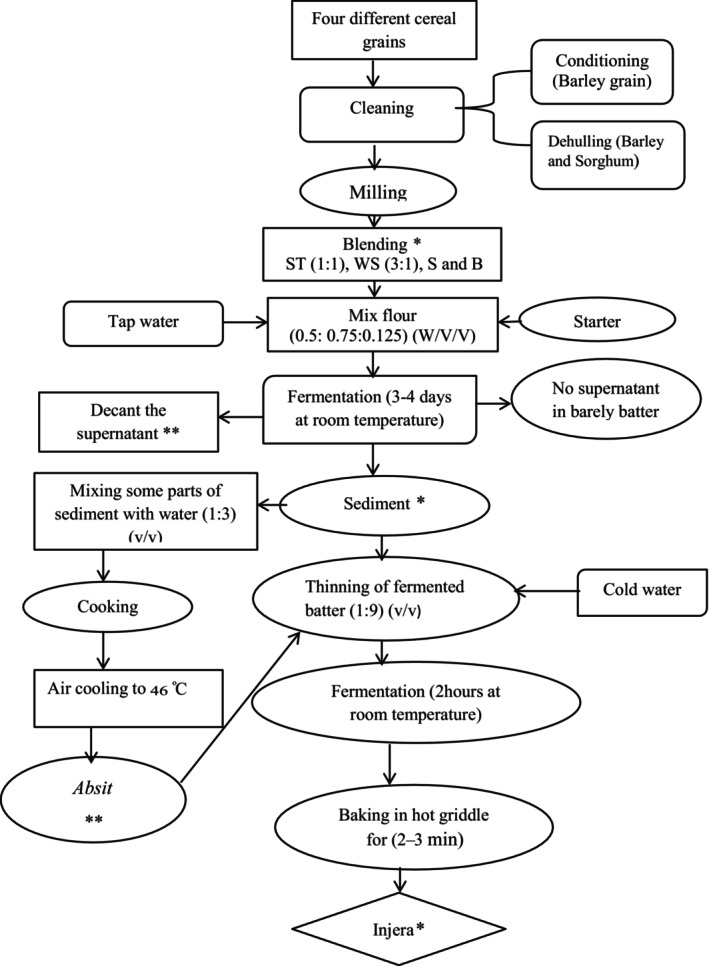
Description of processing of *injera made with different cereal blends and using* different starters. ST = sorghum and tef flour (1:1), WS = wheat and sorghum flour (3:1), S = sorghum flour (100%), and B = barley flour (100%). *Indicates samples were taken for folate analysis. **Indicates no *absit* preparation and decantation process during barely fermentation. Starters: *Ersho* (traditional backslopping) Folate‐producing *L. plantarum* P2R3FA.

The traditional *injera*‐making process was developed after detailed observations from 10 selected households. Households were selected deliberately, where blended cereals are used for *injera* making, and this resulted in a flow diagram presented in Figure 1. Briefly, the dough was prepared by using blended and/or whole flour of 0.5 kg, 0.75 L sterile tap water, and 125 mL starter culture (0.5:0.75:0.125) (w/v/v). The 125 mL starter culture was prepared by mixing 62.5 mL saline containing the *L. plantarum* strain and 62.5 g flour (1:1) (v/g); similar proportion was also used for *ersho* starter. Thereafter, 200 mL sterile tap water was carefully added to cover the surface of the batter and incubated for 4 days at room temperature (first‐phase fermentation). After 4 days of fermentation, liquid present on top of the batter was decanted and replaced by a similar amount of fresh tap water except for barley fermentation where there was no supernatant. Before baking *injera* from the fermented batter, 1/11th of the fermented batter was mixed with sterile tap water (1:3) (v/v), boiled for about 10 min, and cooled to a temperature of around 46°C. The resulting product (*absit*) was added back to the remaining fermented batter to enhance proper fermentation. In case of barley fermentation, sterile cold water was used instead of *absit*. The batter was allowed to ferment for 2 h at 25°C until gas production was observed (second‐phase fermentation). Finally, the fermented liquid batter (450 mL) was poured onto a hot clay with cover and was baked for 2 min to make the final product, *injera*. Three baking were performed for each inoculum mentioned under Section 2.3.1.

#### 2.3.3 Sampling

During *injera* preparation, pH of the batter was measured initially before fermentation and after second‐stage fermentation. Fermented batter was sampled to measure dry matter (DM) and folate contents before the addition of *absit*, and *injera* was also sampled to measure DM and folate contents and subjected to sensory analysis. DM was analyzed by oven drying at 105°C. pH was measured using an aliquot of dough immediately after diluting it with deionized water (1:1) (v/v).

### 2.4 Folate measurement and effect of processing

#### 2.4.1 Folate measurement

The total folate content of tef flour, dough, and *injera* was determined in triplicates using the reference microbiological method after trienzyme extraction (Kariluoto et al., 2004; Tamene, Baye, et al., 2019). The total folate content was determined based on the growth of folate‐dependent strain, *Lactobacillus rhamnosus* ATCC 7469 as the test organism, and (6S)‐5‐formyltetrahydrofolate (5‐HCO‐H4 folate) as the calibrant. Performance of the method was checked by testing a blank sample and certified reference material (BCR‐121 whole meal flour). Only folate contents in the range of certified value (500 ± 70 ng/g dry matter) were accepted. In addition, for triplicate samples, folate content variations of >10% were not accepted. Analytical procedures were carried out under yellow or subdued light. Alternatively, samples and calibrants were covered with aluminum foil. Sample extracts were kept under nitrogen atmosphere whenever necessary.

#### 2.4.2 Effect of processing

The effect of cooking (baking) and fermentation on the total folate content was evaluated and results were expressed as percentage retention.

### 2.5 Contribution of *injera* consumption to the recommended nutrient intake (RNI) of folate

Based on the existing data on *injera* consumption from the Ethiopian National Food Consumption Survey as indicated in Tamene, Kariluoto, et al. (2019) and daily folate requirements (required or recommended nutrient intakes [RNI]) from World Health Organization (WHO, 2004), the contribution of consumption of *injera* made with different cereals to the RNI of folate was estimated for children and women of reproductive age. These population groups were selected because they represent a higher risk for folate deficiency.

### 2.6 Sensory evaluation

The aim of this experiment was to determine whether the folate‐enriched *injera* made with different cereals is acceptable or not by potential consumers. The acceptability and sensory profile for the prepared *injera* were rated by 30 adult healthy volunteers (women of reproductive age), using a 9‐point hedonic scale. The 30 judges of adult healthy volunteers were randomly selected and were provided with the randomly sequenced samples for testing. They were asked to evaluate the products for color, taste, odor, texture (mouth feel), and overall acceptability. As described in Tamene et al. (2022), the panelist used a 9‐point hedonic scale to evaluate the acceptability of *injera*, where 1 = liked extremely and 9 = disliked extremely. They were instructed to cleanse their mouth with water before testing the next sample.

### 2.7 Statistical analysis

Statistical analysis of folate and sensory acceptability was computed using SPSS version 25 software. The folate analyses were carried out in triplicate and the average values and standard deviations were calculated. The differences between means of folate values in different cereal flour, dough, and *injera* as well as differences in acceptability ratings were evaluated using analysis of variance (ANOVA) and Tukey's post hoc tests. Mean differences were considered statistically significant for *p* values ≤.05.

## 3 RESULTS

### 3.1 pH of the dough

The pH of the initial dough made with four different blended cereals recorded at 25°C ranged from 5.5 to 6.1 with an average of 5.8 ± 0.3. After fermentation, the pH value of doughs made with different cereals and fermented with *L. plantarum* ranged from 3.38 to 3.43 with an average value of 3.36 ± 0.07, whereas the pH value of *ersho*‐fermented doughs made with different cereals ranged from 3.30 to 3.42 with an average value of 3.41 ± 0.09. There was no significant difference in pH value of all doughs regardless of the cereal types and starters used.

### 3.2 DM content of flour, dough, and *injera*

The DM content of flour, dough, and *injera* made with different cereals and starters were analyzed and results are shown in Table 2. The average DM of different flour ranged from 91.7 ± 0.57% to 92.7 ± 0.57% by mass. There was no significant difference in DM content of all the flours.

**TABLE 2:** DM content of flour, dough, and *injera (% by mass).*

Blends	Flour	Dough fermented with		*Injera* fermented with	
		*Ersho*	*L. plantarum*	*Ersho*	*L. plantarum*
Tef and sorghum (1:1)	91.7 ± 0.57^a^	39.8 ± 0.15^b^	38.6 ± 0.43^a^	45.3 ± 0.26^b^	43.5 ± 0.3^b^
Wheat and sorghum (3:1)	91.7 ± 0.57^a^	39 ± 0.93^b^	39.5 ± 0.35^a^	41.9 ± 0.53^a^	43.5 ± 0.2^b^
Sorghum (100%)	92.7 ± 0.57^a^	40.8 ± 0.25^b^	41.5 ± 0.35^b^	38.2 ± 0.76^a^	44.7 ± 0.5^b^
Barley (100%)	92.7 ± 0.57^a^	35.6 ± 0.31^a^	37.9 ± 0.94^a^	37.2 ± 0.53^a^	39.9 ± 0.4^a^

*Note*: Values in the table are mean and standard deviation of triplicate samples. Different superscript letters along the column indicate a statistically significant difference at *p* < .05.

The average DM contents of *ersho*‐fermented dough ranged from 35.6 ± 0.31% to 40.8 ± 0.25% by mass. For *L. plantarum*–fermented dough, the DM was in the range of 37.9 ± 0.94% and 41.5 ± 0.35% by mass. Significantly higher DM (41.5 ± 0.35% by mass) was observed for dough made with sorghum, whereas there was no significant difference in DM content between the doughs made from the other cereals.

The average DM content of *injera* made with *ersho* ranged from 37.2 ± 0.53% to 45.3 ± 0.26% by mass. With the exception of *injera* made with tef and sorghum blend, which had significantly higher DM (45.3 ± 0.26), there was no significant difference in DM contents for all the other *injeras* fermented with *ersho*. Regarding *injera* fermented with *L. Plantarum*, the average DM content ranged from 39.9 ± 0.4% to 44.7 ± 0.5% by mass. The DM of *injera* made from barley (39.9 ± 0.4% by mass) was significantly lower than the *injera* made from other cereals.

### 3.3 Folate content of flour, dough, and *injera* made with different cereals and fermented with different starters

The average folate contents of different blends of flour ranged from 32.2 ± 1.5 to 49.9 ± 1.1 μg/100 g (Table 3). Significantly higher folate content (49.9 ± 1.1 μg/100 g) was recorded in sorghum flour, whereas significantly lower folate content (32.2 ± 1.5 μg/100 g) was observed in barley flour. There was no significant difference between average folate content of tef and sorghum (1:1)– and wheat and sorghum (3:1)–blended flour, which had 42.6 ± 1.4 and 40.4 ± 1.5 μg/100 g of folate, respectively.

**TABLE 3:** Folate content (μg/100 g) of flour, dough, and *injera* fermented with *L. plantarum* and *ersho*, DM basis.

Blends	Flour	Dough fermented with		*Injera* fermented with	
		*Ersho*	*L. plantarum*	*Ersho*	*L. plantarum*
Tef and sorghum (1:1)	42.6 ± 1.4^b^	29.1 ± 0.9^aB^	53.5 ± 3.5^bC^	17.5 ± 0.7^aA^	30.2 ± 0.9^aB^
Wheat and sorghum (3:1)	40.4 ± 1.5^b^	32.2 ± 1.3^bcB^	56.9 ± 1.6^bcC^	23.6 ± 0.7^bA^	35.4 ± 1.3^abB^
Sorghum (100%)	49.9 ± 1.1^c^	27.6 ± 2.7^aB^	60.1 ± 1.6^cD^	23.4 ± 1.1^bA^	32.3 ± 5.1^aC^
Barley (100%)	32.2 ± 1.5^a^	34.5 ± 0.7^cB^	45.5 ± 1.3^aC^	20.8 ± 0.9^bA^	32.7 ± 1.5^aB^

*Note*: Values are means and standard deviations of triplicate samples. Different lower‐case superscript letters along the column indicate a statistically significant difference at *p* < .05 and different upper case superscript letters across the row indicate a statistically significant difference at *p* < .05.

The average folate contents of different dough fermented with *ersho* ranged from 27.6 ± to 34.5 ± 0.7 μg/100 g. The highest folate content (34.5 ± 0.7 μg/100 g) was recorded in barley batter, however, it was not statistically different from folate of wheat and sorghum–blended dough (32.2 ± 1.3 μg/100 g). The lowest concentration of folate was observed in sorghum batter (27.6 ± 2.7 μg/100 g). However, it was not statistically different from the folate of tef and sorghum–blended dough (29.1 ± 0.9 μg/100 g).

For *L. plantarum* strain‐fermented doughs, the average folate contents were much higher and were in the range of 45.5 ± 1.3 and 60.1 ± 1.6 μg/100 g_._ The highest folate content (60.1 ± 1.6 μg/100 g) was recorded in sorghum batter, followed by wheat and sorghum–blended dough (56.9 ± 1.6 μg/100 g). Significantly lower concentration of folate was observed in *L. plantarum*–fermented barley batter (45.5 ± 1.3 μg/100 g) but was not significantly different from those of wheat and sorghum (3:1)– and sorghum and tef (1:1)‐blended doughs.

The average folate contents of *injera* made with different cereals and fermented using *ersho* ranged from 17.5 ± 0.7 to 23.6 ± 0.7 μg/100 g. Among the different blends, significantly lower folate contents (17.5 ± 0.7 μg/100 g) were observed in tef and sorghum–blended *injera*. However, there was no significant difference in folate contents among the other *injera* samples (*p* ≥ .05).

*L. plantarum–*fermented *injera* made with different cereals had folate content between 30.2 ± 0.9 (tef–sorghum blend) and 35.4 ± 1.3 μg/100 g (wheat–sorghum blend) (Table 3 **)**, with no significant differences between the different *injeras*.

The feasibility of enhancing the folate content of dough and *injera* made with different cereal blends with *L. plantarum* fermentation was evaluated by comparing it to dough and *injera* obtained with *ersho* fermentation. In all cases, doughs fermented with the strain (*L. plantarum*) were found to have significantly higher folate contents than those fermented with *ersho* (Table 3). Looking at specific doughs, tef and sorghum (1:1)–blended dough had 29.1 ± 0.9 and 53.5 ± 3.5 μg/100 g, wheat and sorghum (3:1)–blended dough had 32.2 ± 1.3 and 56.9 ± 1.6 μg/100 g, sorghum dough had 27.6 ± 2.7 and 60.1 ± 1.6 μg/100 g, and barley dough had 34.5 ± 0.7 and 45.5 ± 1.3 μg/100 g folate contents when they were fermented with *ersho* and *L. plantarum* strain, respectively.

Similarly, in all cases, *injeras* fermented with our strain (*L. plantarum*) had significantly higher folate contents as compared to all *injeras* fermented with *ersho* as shown in Table 3. Looking at specific *injeras*, tef and sorghum (1:1)–blended *injera* had 17.5 ± 0.7 and 30.2 ± 0.9 μg/100 g, wheat and sorghum (3:1)–blended *injera* had 23.6 ± 0.7 and 35.4 ± 1.3 μg/100 g, sorghum *injera* had 23.4 ± 1.1 and 32.3 ± 5.1 μg/100 g, and barley *injera* had 20.8 ± 0.9 and 32.7 ± 1.5 μg/100 g folate contents when they were fermented with *ersho* and the strain, respectively.

### 3.4 Effect of fermentation on folate content of dough

**FIGURE 2:**
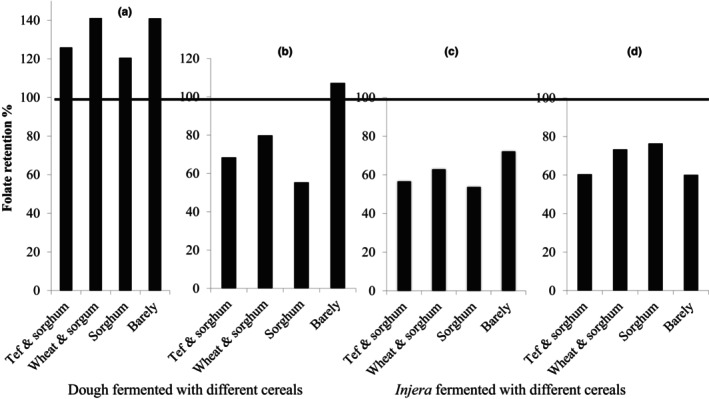
Folate retention (%) due to fermentation and thermal treatment (baking) of *injera*. (a) Folate retention (%) due to fermentation using *L. plantarum*; (b) Folate retention  (%) due to fermentation using *ersho*; (c) Folate retention (%) due to baking of *injera* fermented using *L. plantarum*; (d) Folate retention (%) due to baking of *injera* fermented using *ersho*. Folate retention (%) due to fermentation = Folate dough/Folate flour × 100. Folate retention (%) due to baking = Folate *injera*/Folate dough × 100.

Percentage retention of folate after fermentation was calculated by comparing the amount of folate content in tef flour and dough on DM basis (Figure 2a,b). A value above 100% indicated that fermentation led to increases in folate, whereas retention values less than 100% indicated consumption/losses of folate.

Folate retention after fermentation of different cereals with *L. plantarum* ranged from 120 to 141% **(**Figure 2a **).** This showed more than 100% folate retention for all of the doughs fermented with the strain regardless of the blend used. The highest retention (141%) was recorded in dough made with wheat and sorghum blend (3:1). In contrast, fermentation with *ersho* yielded retention ranging from 68% to 107% (Figure 2b). This showed less than 100% folate retention for all doughs with the exception of barley, which had folate retention of 107%. The higher retention for barley dough may be due to the absence of decantation process which may prevent the losses of folate and/or due to the presence of naturally occurring folate‐producing microorganisms involved in the fermentation process.

### 3.5 Effect of baking on folate content of *injera* made using *ersho* and *L. plantarum*

Regardless of the type of cereal blends and the type of inoculants or starters used for fermentation, retention of folate after baking was less than 100%, with an average of 66% (56% to 76%) for *L. plantarum* (Figure 2c) and 68% (60% to 76%) for *ersho* (Figure 2d).

### 3.6 Folate content and calculated contribution of *injera* made with different cereals and starters to folate requirements (RNI)

The average folate content of *injera* made with different cereals and fermented with *ersho* was in the range of 7.82 ± 0.61 and 9.79 ± 0.28 μg/100 g per fresh weight (FW). The lowest average folate content (7.82 ± 0.61 μg/100 g FW) was recorded in barley *injera* and the highest (9.79 ± 0.28 μg/100 g FW) was observed in wheat and sorghum blend (3:1) *injera* **(**Table 4). However, the average total folate content of *injera* made with different cereals and fermented with *L. plantarum* ranged from 13.13 ± 0.59 to 15.45 ± 0.58 μg/100 g FW. The least folate content (13.13 ± 0.59 μg/100 g FW) was observed in barley and the highest (15.45 ± 0.58 μg/100 g FW) was noted in wheat and sorghum blend (3:1) *injera*.

**TABLE 4:** Contribution of *injera* made with different cereals and starters to folate intake requirements (RNI).

Starters used	*Injera*	Folate (μg/100 g FM)	Contribution to RNI (%)	
			Children (1–3 years)	Women (19 years and older)
*Ersho*	Tef & sorghum (1:1)	7.99 ± 0.34	3.5	4.1
	Wheat & sorghum (3:1)	9.79 ± 0.28	4.3	4.9
	Sorghum	8.94 ± 0.74	3.9	4.5
	Barley	7.82 ± 0.61	3.4	3.9
*L. plantarum*	Tef & sorghum (1:1)	13.23 ± 0.43	5.8	6.7
	Wheat & sorghum (3:1)	15.45 ± 0.58	6.8	7.8
	Sorghum	14.48 ± 2.30	6.3	7.3
	Barley	13.13 ± 0.59	5.7	6.6

*Note*: Values of folate are means ± standard deviation. Average *injera* consumption by children = 66 g/day, and by women of reproductive age = 202 g/day (Tamene, Kariluoto, et al., 2019).

Contribution of *injera* consumption to folate requirements was calculated by estimating folate supplied by the average portion size of *injera* consumed by children (66 g/day) and women of reproductive age (202 g/day) (Tamene, Kariluoto, et al., 2019), relative to the RNI of folate (150 μg/day) for children and (400 μg/day) for women of reproductive age (WHO, 2004). The least folate contribution to RNI was observed in *injera* fermented by traditional process with *ersho*, which contributed 3.4% to 4.3% of the RNI for children and 3.9% to 4.9% of the RNI for women of reproductive age. The highest contributions to the folate RNI to both population groups were observed by wheat and sorghum *injera*, whereas the least contribution was observed for barley *injera*.

Folate content of *L. plantarum–*fermented *injera* was much higher than the *ersho*‐fermented *injera* and contributed to 5.7% to 6.8% of the RNI for children and 6.6% to 7.8% of the RNI for women of reproductive age.

### 3.7 Sensory acceptability of different *injera* made using *L. plantarum* and *ersho*

All of the samples made using different cereals and selected inoculums were found to be in the acceptable range with the exception of sorghum *injera*, which was rated as neither liked nor disliked by the panelists **(**Table 5 **).** The overall acceptability test result showed that sorghum *injera* fermented with both inoculums (*ersho* and *L. plantarum* P2R3FA) was the least acceptable product, whereas tef and sorghum blended and 100% barley *injeras* fermented with both inoculums were the most acceptable *injera*. Looking at each specific parameter, with the exception of color, for all other sensory parameters (taste, odor, texture, and appearance), wheat and sorghum blended and 100% sorghum *injeras* fermented with both inoculums were found to be the least liked product.

**TABLE 5:** Sensory acceptability of different *injera* fermented with *ersho* and *L. plantarum* starters.

Starter	Injera		Color	Taste	Odor	Texture	Appearance	Overall acceptability
*Ersho*	Tef and sorghum (1:1)		2.3 ± 1.3^a^	2.6 ± 1.8 ^a^	2.7 ± 1.6 ^a^	2.9 ± 1.9^a^	2.6 ± 1.6^a^	2.8 ± 1.6^a^
	Wheat and sorghum (3:1)		2.5 ± 1.9^a^	4.3 ± 2.1^c^	4.4 ± 2.1^b^	4.3 ± 2.1^b^	4.4 ± 2^b^	4.3 ± 1.8^b^
	Sorghum (100%)		3.0 ± 1.6^b^	4.2 ± 2.0^c^	4.3 ± 2.1^b^	4.2 ± 2.2^b^	5.1 ± 2.1^b^	4.7 ± 2.1^b^
	Barley (100%)		2.1 ± 1.7^a^	3.4 ± 1.7^b^	3.0 ± 1.7^a^	2.6 ± 1.3^a^	2.4 ± 1.3^a^	2.9 ± 1.4^a^
*L. Plantarum*	Tef and sorghum (1:1)		2.6 ± 1.9^a^	2.4 ± 1.6^a^	2.7 ± 1.2^a^	2.1 ± 0.9^a^	2.3 ± 0.8^a^	2.4 ± 0.9^a^
	Wheat and sorghum (3:1)		2.6 ± 2.1^a^	4.2 ± 1.9^c^	4.2 ± 1.9^b^	4.1 ± 2.4^b^	4.4 ± 2.2^b^	4.2 ± 1.8^b^
	Sorghum (100%)		3.2 ± 1.5^b^	3.7 ± 1.9^b^	4.2 ± 1.9^b^	4.3 ± 2.1^b^	5.1 ± 2.2^b^	4.8 ± 2.1^b^
	Barley (100%)		2.2 ± 1.2^a^	3.2 ± 2.0^b^	3.2 ± 2.0^a^	2.8 ± 2.1^a^	2.5 ± 1.9^a^	2.5 ± 1.4^a^

*Note*: Values are mean of 30 measurements ± standard deviation. Means followed by different letters in the same column are significantly different at *p* < .05. Range is from 1 = extremely liked to 9 = extremely disliked.

## 4 DISCUSSION

In Ethiopia, *injera* is mainly produced from tef, but it is also prepared from other different cereals like barley, sorghum, and wheat either alone or in combination. Few studies focused on investigating the effect of tef *injera* preparation on folate contents and the extent to which folate‐producing strains (*L. plantarum*) can be used to enhance the folate content of *injera*. The current study investigated the effect of *injera* preparation made with different cereals fermented with *ersho* or a folate‐producing *L. plantarum* strain priorly isolated from tef fermentation. We evaluated the sensory acceptability of the resulting *injera* and estimated the contribution to folate requirement.

The current study shows that all of the cereals’ flour used in our study are relatively good sources of folate. The impact of fermentation (one of the steps in *injera* preparation) was highly variable. In the case of doughs fermented with *L. plantarum*, although highly variable, fermentation leads to net folate production. However, doughs fermented with the traditional process using *ersho* led to reduction of folate with the exception of barley dough which may be due to the presence of other naturally occurring folate‐producing microorganisms and/or the lack of decantation process that may lead to folate loses. In contrast with fermentation, baking invariably led to folate losses, which is in line with findings from Tamene, Kariluoto, et al. (2019).

Recent studies showed that fermented foods such as cereals and dairy products have the potential to increase folate intake and potentially prevent adverse effects related to folate deficiencies (Rollán et al., 2019; Saubade et al., 2017). Our study has also revealed the possibility of increasing folate through fermentation as illustrated, for example, by the greater than 100% folate retention observed in barley fermentation. This result suggests that folate‐producing microorganisms may dominate those that do not produce or consume folate as previous studies confirmed the co‐occurrence of both folate‐producing and consuming microorganisms during fermentation process (Okoroafor et al., 2019). The type of microorganisms and the overall conditions that led to folate production in barley fermentation need further detailed examination. Fermentation of all cereals using *L. plantarum* always led to net folate production, suggesting that the strain is as effective in increasing folate during *injera* fermentations of other cereals as in tef fermentation.

As baking is the second major step in *injera* preparation, any folate that is originally present in a food or produced as a result of fermentation needs to survive the baking temperature to have meaningful health impact. Folate is known to be heat sensitive and cooking/baking conditions can cause varying levels of losses (Lešková et al., 2006). Folate losses due to baking are quite common and have been reported by different scholars (Kariluoto et al., 2004; Lešková et al., 2006; Tamene, Kariluoto, et al., 2019). However, some examples of baked *injera* (*ersho*‐fermented 100% sorghum *injera* and *L. plantarum*–fermented 100% barley *injera*) retained more than 75% of the folates. This finding suggests that folate losses can be minimized if *injera* baking process is optimized.

Based on portion size estimates obtained from the national food consumption survey, the average folate content in our *ersho*‐fermented *injera* samples made from different cereals contributes to less than 5% of the folate RNI for children (1–3 years) and woman of reproductive age. Use of the same cereals with equivalent proportions but changing the inoculum (*L. plantarum* instead of *ersho*) yields *injera* with better folate contents, which can contribute to more than 5% of the daily folate requirement of both women and young children. Assuming that two servings (∼350 g/serving) of *injera* are consumed per day by women of reproductive age, *injera* made from cereals other than tef could contribute to more than 10% of the RNI if *L. plantarum* is used as inoculum. The present study clearly showed that all *injera* prepared using the traditional process had the lowest folate content as compared to its counterpart process which used *L. plantarum*.

The contribution of consumption of these *injeras* made using *L. plantarum* to folate requirement is a bit lower than tef *injera* fermented with the same strain (Tamene et al., 2022). This could be obviously due to differences in the initial total folate content where significantly higher initial total folate is observed in tef flour (Tamene, Kariluoto, et al., 2019) as compared to flours of any cereals used in this study. Otherwise, in both cases, the strain *L. plantarum* is found to be effective in enhancing the folate content by doubling the contribution of *injera* relative to the traditional process which uses *ersho* as inoculum.

Current studies have focused on folate improvement through fermentation with minimum emphasis on the organoleptic quality of the final product, which is one of the quality parameters for the food to be consumed. In the present study, almost all *injera* samples fermented with the *L. plantarum* strain were accepted by the panelists with the exception of 100% sorghum *injera*, whose test is less familiar in urban settings.

The food industry is currently working to develop new balanced and healthy diets to meet growing consumer demand. Therefore, a lot of research has been done to create new nutritional products with better or equal organoleptic properties than regular products. Consequently, the use of microorganisms such as LAB can play an important role by producing essential nutrients such as folate or by acting as probiotics (Falah, Vasiee, et al., 2021; Falah, Zareie, et al., 2021).

Certain limitations need to be considered when interpreting our findings. To what extent discarding of the supernatants contributed to folate loss was not quantified which would have enabled us to be clear with the cause of folate losses during fermentation (material loss or loss due to consumption by microorganisms). The study used a fixed proportion during blending of cereals but multiple proportions could yield a different result both on the folate content and sensory attributes of the final product. The present study focused only on few common cereals as there could be other cereals that can be used for making *injera* in other parts of the country.

## 5 CONCLUSION

Cereals such as wheat, barley, sorghum, and tef could be considered as sources of folate, and fermenting it to prepare *injera* can either increase or decrease its folate content, while baking always leads to folate losses. Relative to *injera* fermentation with *ersho*, our *L. plantarum* strain almost always led to increases in folate content during fermentation and less loss during baking of the doughs. Consequently, *injeras* fermented with *L. plantarum*, irrespective of the type of cereal used, led to higher contribution to daily folate intake than *injera* fermented with *ersho*. Our study has clearly indicated that *L. plantarum* could be used for the development of fermented foods like *injera* prepared with different cereal types with better folate content and consumer preferences. This research also showed that *L. plantarum* strain can be used as a potential folate source not only for *injera* made with tef but also for any *injera* that could be made with other cereals. Further studies on safety characterization of the strain like antibiotic profile activity are recommended.

## AUTHOR CONTRIBUTIONS

**Aynadis Tamene:** Conceptualization (equal); formal analysis (equal); investigation (equal); methodology (equal); resources (equal); supervision (equal); validation (equal); writing – original draft (equal); writing – review and editing (equal). **Kaleab Baye:** Conceptualization (equal); methodology (equal); project administration (equal); resources (equal); supervision (equal); validation (equal); writing – review and editing (equal). **Tesfaye Mekuriyaw:** Formal analysis (equal); funding acquisition (equal); investigation (equal); writing – original draft (equal).

## FUNDING INFORMATION

This work was partially supported by the DARE project, funded by the European Commission, DESIRA partnership for innovation program.

## CONFLICT OF INTEREST STATEMENT

The authors declare no conflict of interest.

## ETHICS STATEMENT

This study was approved by the Institutional Review Board of the College of Natural and Computational Sciences of Addis Ababa University.

## INFORMED CONSENT

Written informed consent was obtained from all study participants for the sensory analysis.

## Data Availability

The data that support the findings of this study are available on request from the corresponding author. The data are not publicly available due to privacy.
